# Design and operation of a Peucedani Radix weeding device based on YOLOV5 and a parallel manipulator

**DOI:** 10.3389/fpls.2023.1171737

**Published:** 2023-05-30

**Authors:** Xuechen Zhang, Chengmao Cao, Kun Luo, Zhengmin Wu, Kuan Qin, Minhui An, Wuyang Ding, Wang Xiang

**Affiliations:** ^1^School of Engineering, Anhui Agricultural University, Hefei, China; ^2^School of Tea and Food Science and Technology, Anhui Agricultural University, Hefei, China; ^3^State Key Laboratory of Tea Plant Biology and Utilization, Hefei, China

**Keywords:** Peucedani Radix, YOLOv5, trajectory planning, precision weeding, parallel manipulator

## Abstract

To avoid excessive use of herbicides in the weeding operations of Peucedani Radix, a common Chinese herb, a precision seedling avoidance and weeding agricultural robot was designed for the targeted spraying of herbicides. The robot uses YOLOV5 combined with ExG feature segmentation to detect Peucedani Radix and weeds and obtain their corresponding morphological centers. Optimal seedling avoidance and precise herbicide spraying trajectories are generated using a PSO-Bezier algorithm based on the morphological characteristics of Peucedani Radix. Seedling avoidance trajectories and spraying operations are executed using a parallel manipulator with spraying devices. The validation experiments showed that the precision and recall of Peucedani Radix detection were 98.7% and 88.2%, respectively, and the weed segmentation rate could reach 95% when the minimum connected domain was 50. In the actual Peucedani Radix field spraying operation, the success rate of field precision seedling avoidance herbicide spraying was 80.5%, the collision rate between the end actuator of the parallel manipulator and Peucedani Radix was 4%, and the average running time of the parallel manipulator for precision herbicide spraying on a single weed was 2 s. This study can enrich the theoretical basis of targeted weed control and provide reference for similar studies.

## 1 Introduction

Peucedani Radix (Chinese name Qianhu), the dried root of *Peucedanum praeruptorum* Dunn (The State Pharmacopoeia Commission of P.R. China, 1997; Pharmacopoeia, 2010; The State Pharmacopoeia, C., 2010), is a common Chinese herb. Peucedani Radix has been widely used for centuries to treat colds and coughs (Chang et al., 1986). Competition for water, nutrients, space, and sunlight between weeds and Peucedani Radix (Li et al., 2021) significantly reduces the yield of Peucedani Radix, causing huge economic losses. However, the main economic value of Peucedani Radix lies in its buried rhizome, which mechanical weeding operations can damage along with plant stems (Quan et al., 2022). At present, Peucedani Radix weeding is mainly achieved using sprayed herbicides; however, spraying herbicides over a large area on water, air, and soil can lead to environmental problems (Villette et al., 2022). Therefore, reducing the use of herbicides and ensuring the yield of Peucedani Radix is a major challenge. Precision seedling avoidance spraying is an effective way to maintain the use of herbicides and effectively control weeds in Peucedani Radix fields (Özlüoymak, 2022).

The prerequisite for accurate seedling avoidance spraying of herbicides is accurate spraying without damaging the crop. Some researchers have proposed distinguishing weeds from crops using features such as color space, leaf texture, spectrum characteristics, and morphological size (Hamuda et al., 2017; Strothmann et al., 2017; Sujaritha et al., 2017; Zheng et al., 2017). However, the performances of these methods are influenced by a complex variety of factors, including weed density, light conditions, crop–weed overlap, weather, and crop growth stage. Therefore, an efficient and stable algorithm is needed to handle the complex and diverse field operation situations (Zou et al., 2021). In recent years, deep learning techniques have developed rapidly. Chavan and Nandedkar (2018) combined Alexnet and Vggnet models to form the AgroAVNET network for classification of crops and weeds. Dos Santos Ferreira et al. (2017) classified soybean and weeds using ConvNets. Tang et al. (2017) used K-means combined with Convolutional Neural Network (CNN) to identify and classify weeds. Although the accuracy of these classification methods is relatively high, the operation requires the segmentation of crops and weeds, and the classification time of a single image will increase with the number of weeds and crops in the image. Picon et al. (2022) achieved semantic segmentation of multiple weed and maize crops using Dual Pyramid Scene Parsing Network (PSPNet). Quan et al. (2019) used an improved Fast Region-based Convolutional Network (Fast-RCNN) model with Visual Geometry Group 19 (VGG19) to achieve maize seedling detection at different growth stages and under various weather conditions. Ahmad et al. (2021) and Quan et al. (2022) used the You Only Look Once Version 3 (YOLOV3) network model to detect and classify common weeds in maize fields and the results showed that the average detection accuracy of YOLOV3 was above 93% in all cases. Although these methods have high identification accuracy in actual field operations, they require extensive labeling of weeds and crops, which greatly increases the workload of detection (Hasan et al., 2021; Li et al., 2022).

It is challenging to use an end-effector to precisely spray herbicide onto weed surfaces without collisions between the end-effector and crop (Li et al., 2022). Utstumo et al. (2018) designed an Asterix autonomous robotic platform that enables drop-on-demand spraying of herbicides from the top to the bottom of the crop through nozzles with a lateral spacing of 6 cm. Partel et al. (2019) designed precision spraying systems adapted to crop row spacing. Villette et al. (2021; 2022) compared different nozzle spray shapes, nozzle spacing, and six spraying strategies to obtain the optimal pattern of triangular-shaped sprays combined with overlapping sprays, which significantly reduced the amount of herbicide used. However, the above method cannot avoid potential damage to the crop if the nozzles are too high due to improper nozzle spacing and height setting, causing the herbicide to be sprayed onto the surface of the crop during equipment travel.

A key objective of this research was to design an algorithm that identifies crops and weeds quickly and accurately, while reducing the workload of dataset production. Another important goal was to ensure that the spray actuation equipment avoids crop injury during accurate herbicide spraying. To achieve these two goals, this study developed an intelligent Peucedani Radix weeding agricultural robot, which uses You Only Look Once Version 5 (YOLOV5) with Extra-Green (ExG) feature segmentation for crop and weed recognition, and a parallel spraying device with Particle Swarm Optimization (PSO)-Bezier seedling avoidance trajectory for herbicide spraying. By applying YOLOV5 for crop identification and ExG feature segmentation for weed identification, the crop and weed identification problem is transformed into a binary problem, thus simplifying the complex weed labeling work. In addition, the PSO-Bezier curve is used to achieve accurate seedling avoidance spraying of herbicides based on crop characteristics to reduce pesticide usage and achieve seedling avoidance during operation, which significantly reduces the amount of pesticide residues on the crop surface and energy consumption.

## 2 Materials and methods

### 2.1 System overview

**Figure 1:**
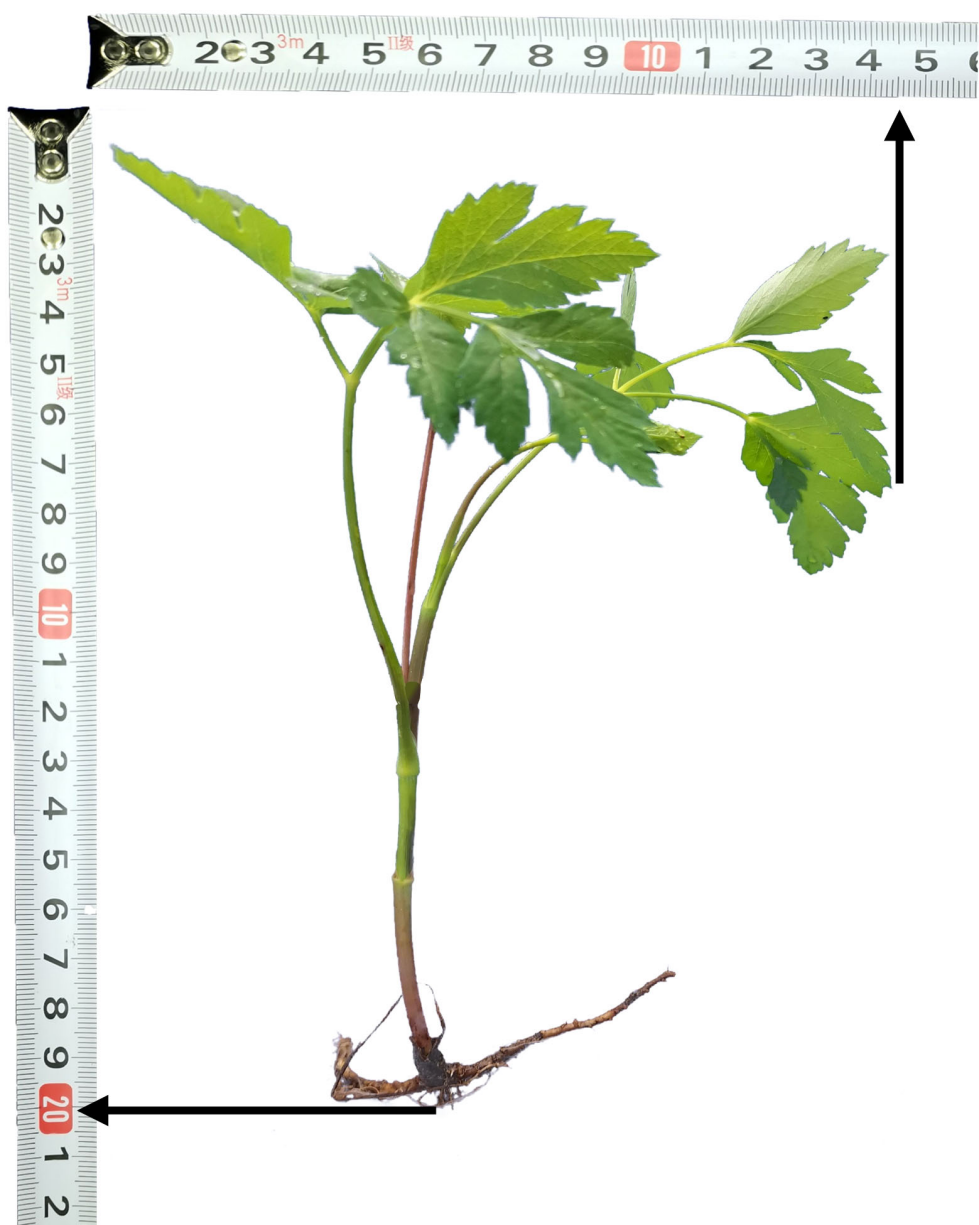
Semiannual morphological parameters of Peucedani Radix growth.

**Figure 2:**
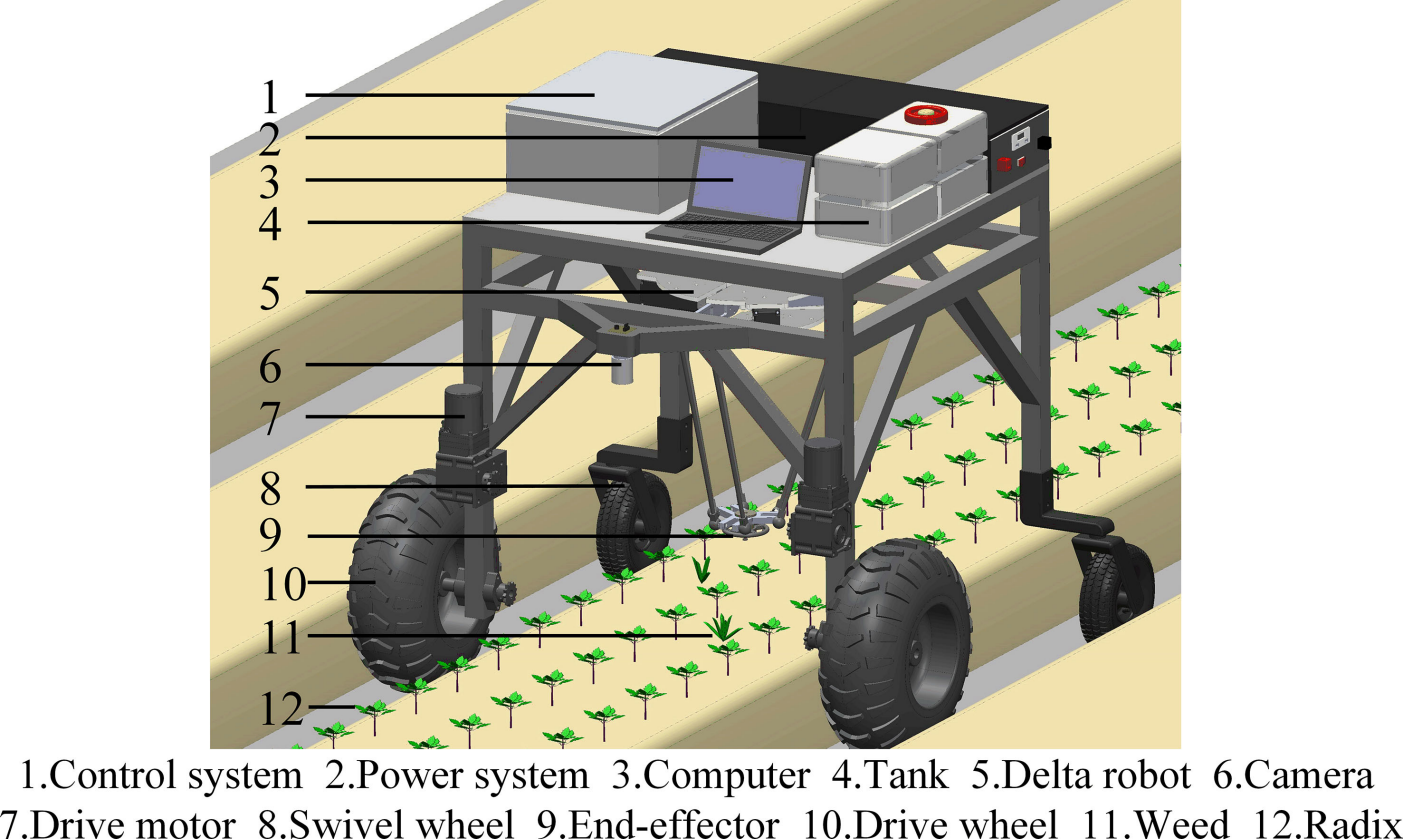
Schematic of the intelligent Peucedani Radix weeding agricultural robot structure.

**Figure 3:**
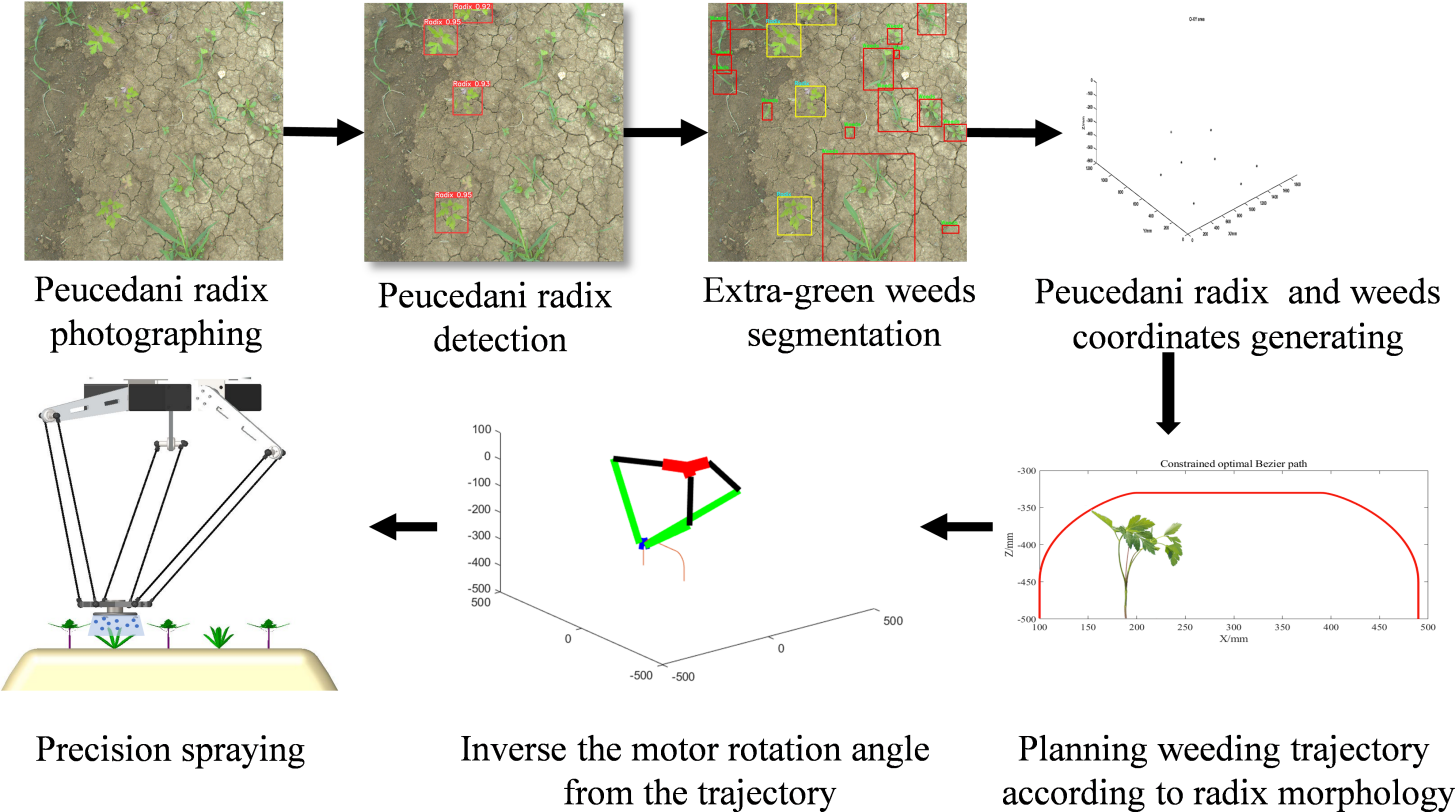
Schematic of the Peucedani Radix weeding process.

The biology of Peucedani Radix seedlings is characterized by an erect growth type (**Figure 1**). Therefore, to facilitate subsequent studies, the morphology of Peucedani Radix plants was simplified in this study as cylinders of different diameters. An intelligent Peucedani Radix weeding agricultural robot was designed, as shown in **Figure 2**. The agricultural robot is driven by Direct Current motors and is equipped with parallel robotic arms and circular nozzles on the end-effectors. The crop and weeds on the field ridge are photographed by a camera mounted at 90° to the horizontal and the locations of Peucedani Radix and weeds are identified in real time by a computer. The computer performs PSO-Bezier trajectory planning for the robotic arm end-effector based on the position and morphological parameters of Peucedani Radix and the position of the weed to achieve precise seedling avoidance for herbicide spraying. The corresponding workflow schematic is shown in **Figure 3**.

### 2.2 Image dataset construction

The growth cycle of Peucedani Radix plants used in this study was one year, planted in December 2021. Peucedani Radix fields are usually weeded when the plants are approximately 15 cm tall. Peucedani Radix planted in the Nongcui Garden of Anhui Agricultural University, Hefei, Anhui Province, China (117°14’E, 31°52’N) were photographed in April, June, and August 2022 to produce the dataset. A Balser industrial camera (acA1920-150uc, Germany) was used for image acquisition, cropping the left and right unrelated areas of the image to improve acquisition speed. In total, 5,092 images with a resolution of 1200 × 1200 were collected. Since the dataset pictures were taken at 1 s intervals in a cycle, the differences between adjacent pictures were not obvious. In addition, some pictures of poor quality were obtained during the dataset acquisition process and these pictures could not meet the experimental requirements. Therefore, it was necessary to filter the dataset manually. Finally, 2,347 images were selected as the dataset and the dataset was enhanced by changing the brightness and darkness of the images, mirroring, and other adjustments to improve the richness of the sample. In contrast to other dataset annotations, this annotation only labeled Peucedani Radix plants. Finally, the dataset of 2,347 images was expanded to 9,388 images and the enhanced dataset was divided into a training set and a validation set at a 4:1 ratio (**Table 1**).

**Table 1:** Main parameters of YOLOV5 network dataset.

Name	Quantity (No.)	Peucedani Radix number (No.)
Preferred dataset	2,347	4,823
Image augmentation	9,388	19,297
Training dataset	7,577	15,439
Test dataset	1,811	3,858

### 2.3 Crop and weed identification

**Figure 4:**
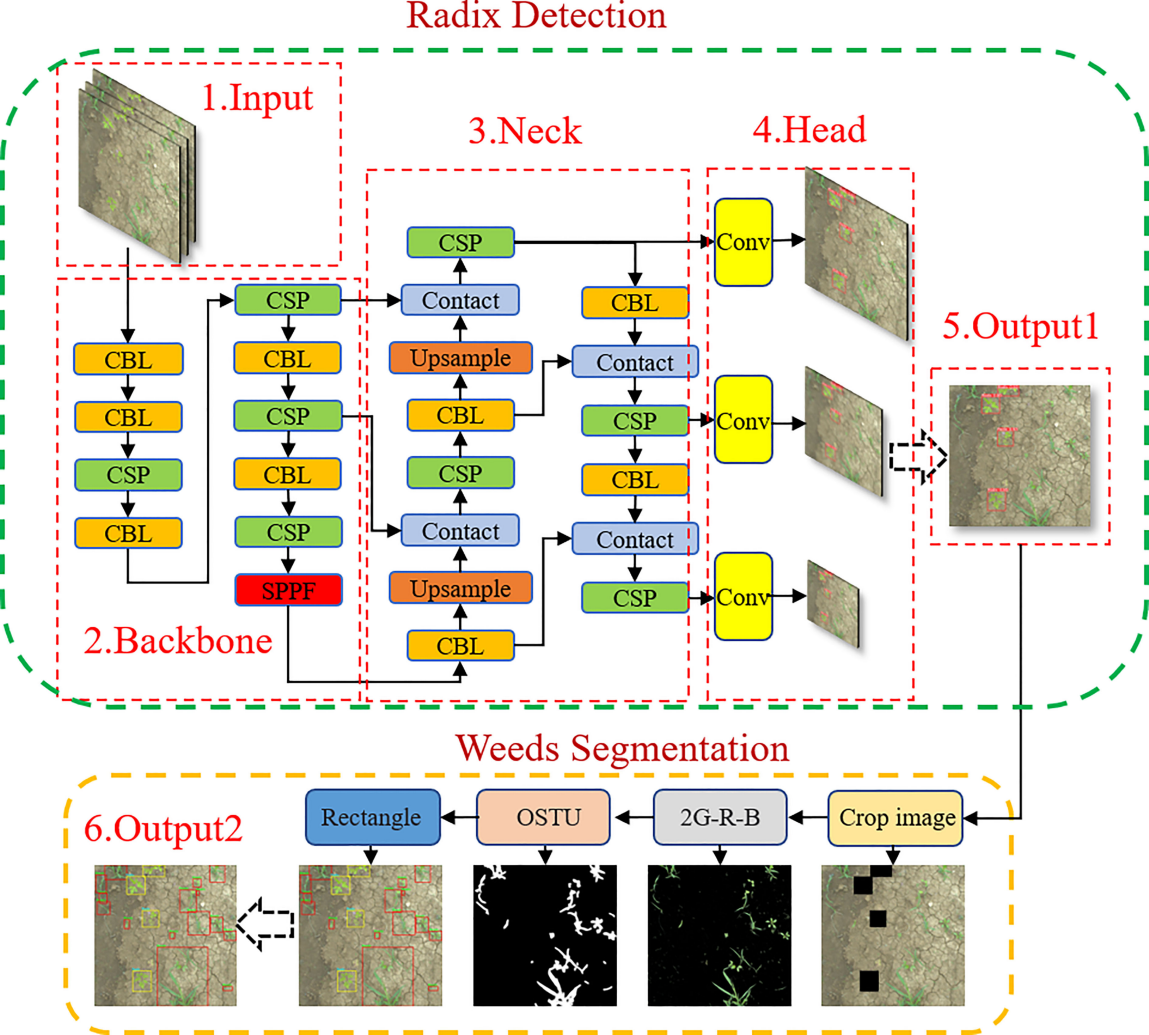
Crop and weed identification model structure.

Since the YOLO network is currently one of the best-performing algorithms in the target detection field, this study used the fast and accurate YOLOV5 network combined with ExG feature segmentation to detect crops and weeds. The structure of the crop and weed detection model is shown in **Figure 4**, which is mainly divided into two parts: Peucedani Radix detection and weed segmentation. The Peucedani Radix detection component consists of the YOLOV5 network, which was developed from the previous YOLOV4 and YOLOV3 (Redmon and Farhadi, 2018) networks. The YOLOV5 network is divided into three parts: Backbone, Neck, and Head. Compared with that of YOLOV4, the first layer of the Backbone network in YOLOV5 has an additional 6 × 6 sized convolutional layer. In the Neck part, YOLOV5 uses Spatial Pyramid Pooling – Fast (SPPF) network structure, compared to the previous version which uses Spatial Pyramid Pooling (SPP) structure. SPPF modifies the 9 × 9 and 13 × 13 sized MaxPool layers into two and three 5 × 5 sized MaxPool layers, respectively. The modified network achieves the same result but is two times faster. The weed segmentation component is composed of four parts: crop image, 2G-R-B (ExG feature segmentation), maximum between-cluster variance (OSTU), and rectangle.

In this study, the YOLOV5 network model was first used to process the images captured by the camera to determine the locations of the Peucedani Radix plants (Output1). The Peucedani Radix plants were then separated from the original image by cropping the minimum external rectangular box. Next, the cropped image was fed into the 2G-R-B (ExG) algorithm to obtain the foreground image of the weed after separation of the weed from the soil. Subsequently, the foreground image of the weed was grayed out and adaptive binarization was implemented using OSTU. The connectivity domain of the binarized image was processed by operations such as erosion expansion to obtain a reasonable weed connectivity domain. Finally, morphological center extraction and minimum outer rectangle drawing were performed on the weed connectivity domain to detect the weed and Peucedani Radix plants and their corresponding locations.

The training platform used a host containing an Intel Core i7-11700F (2.5 GHz) octa-core CPU, an NVIDIA RTX3060 (1,876 MHz) GPU, and 32 GB of RAM, running on Windows 10. The software tools included CUDA 11.4, CUDNN 8.2.2, and Python 3.8 and the experiments were implemented in the Pytorch framework.

### 2.4 Weed trajectory planning

YOLOV5 combined with the ExG feature segmentation algorithm was used to detect Peucedani Radix and weeds and obtain their coordinate information under the robot coordinate system. To avoid collision of the end-effector of the robot arm with Peucedani Radix plants, an optimal motion path needs to be found for the specified coordinates of the start and end points of the end-effector motion to achieve efficient seedling avoidance and weed spraying.

**Figure 5:**
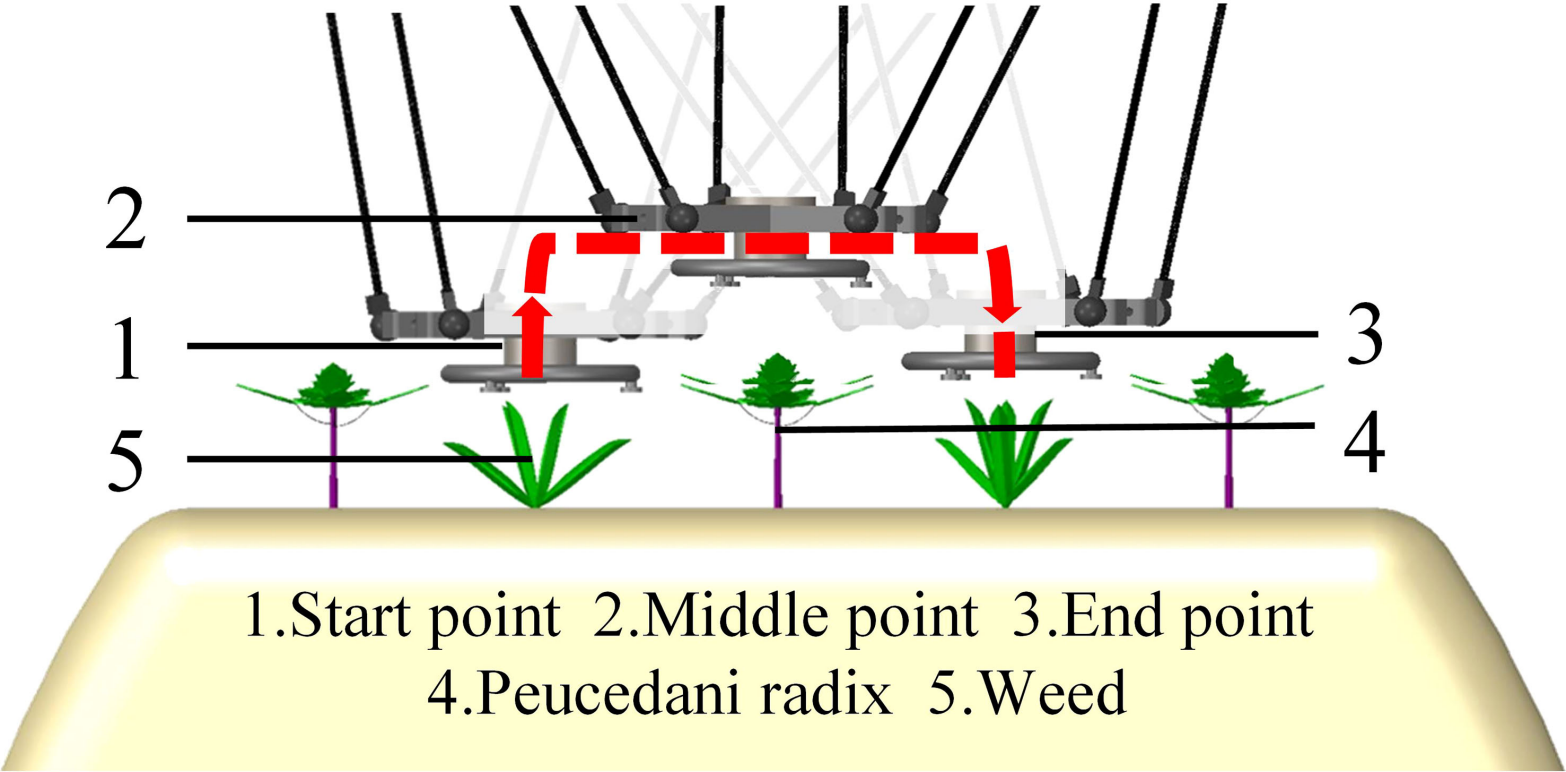
Schematic of end-effector weeding trajectory. Schematic diagram of the seedling avoidance and weeding trajectory of the end-effector.

The process of moving the end-effector from the current weed position to the next weed position is first defined as a weeding cycle. As shown in the red curve in **Figure 5**, the trajectory of the end-effector was designed in one weeding cycle and the center of the end-effector moves along the curve to achieve precise weeding and avoid spraying herbicide onto the crop surface. To reduce the overall vibration of the robotic arm during the transition phase and crop avoidance, Particle Swarm Optimization and third-order Bezier curves combined with crop morphology parameters were used to generate the optimal transition trajectory for the end-effector movement in the vertical to horizontal direction.

**Figure 6:**
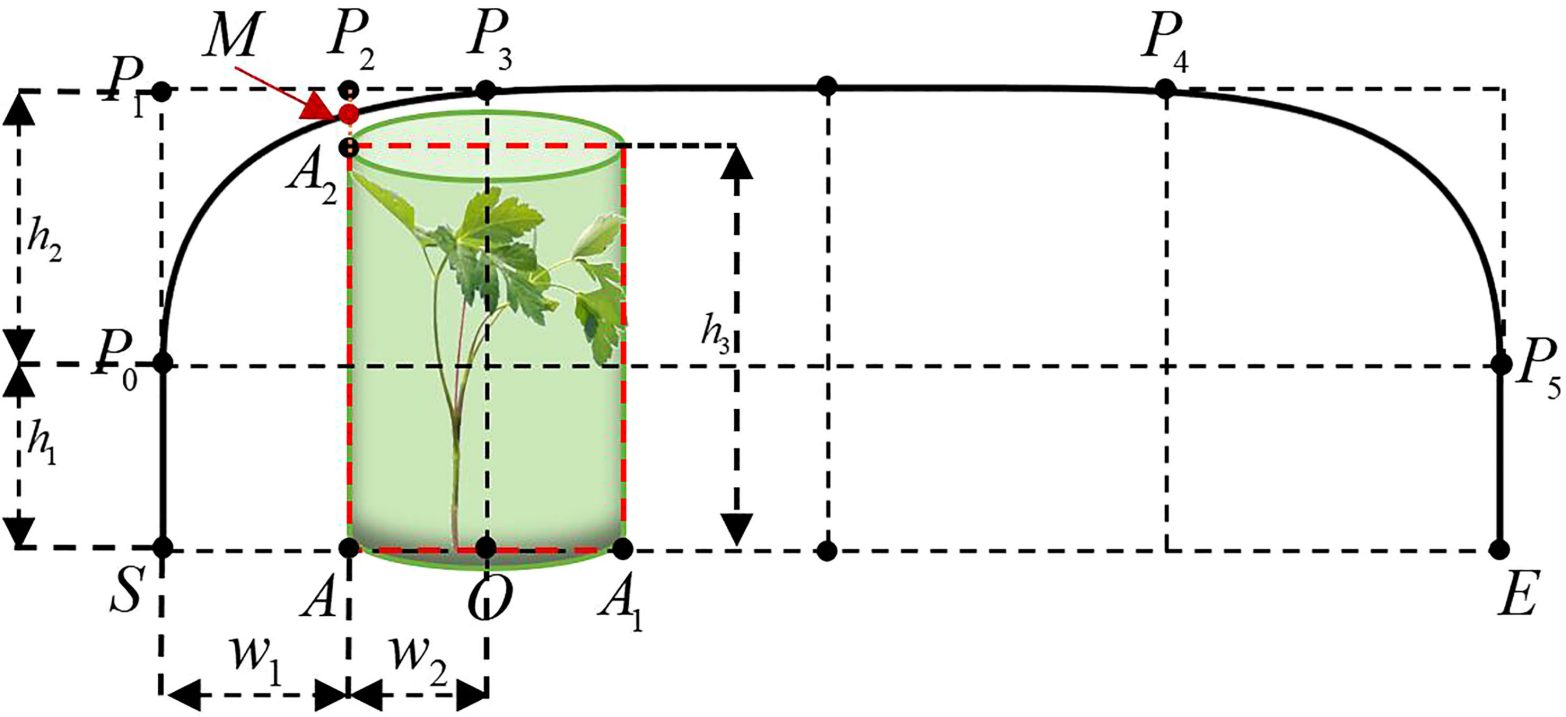
Bezier weeding trajectory execution paths and their corresponding control points.

As shown in **Figure 6**, S and E are the starting and ending points of the trajectory, respectively, corresponding to the coordinates of the weed. The “∩” type trajectory of seedling avoidance and weeding was established in the vertical plane N of the SE line segment (Yang et al., 2021). For analysis, the plane N was rotated to the O-XZ plane, and the point S was set as the origin. To facilitate the calculation, a Peucedani Radix plant was regarded as a cylinder with constant height and changing diameter and the height $h_{2}$ of the cylinder was set to 150 mm using numerous statistics. The radius of the cylinder was set to $w_{2}$ and the distance from the center of the cylinder bottom circle to the point S was $w_{1}+w_{2}$ . $S\to P_{0}$ is the ascent phase with height $h_{1}$ , $P_{0}\to P_{1}\to P_{2}\to P_{3}$ is the transition phase designed using a third-order Bezier curve with height $h_{2}$ , $P_{3}\to P_{4}$ is the horizontal shift phase, $P_{4}\to P_{5}$ is the transition phase designed using a third-order Bezier curve, and $P_{5}\to E$ is the descent phase. In **Figure 6**, $P_{0},P_{3}$ are the starting and ending points of the Bezier curve, respectively, and $P_{1},P_{2}$ are the first and second control points, respectively. The shape of the third-order Bezier curve is adjusted by adjusting the position of the $P_{1},P_{2}$ points to ensure that the end-effector of the robot arm avoids the crop as it moves along the Bezier curve to the M point.

The Bezier curve equation is as follows:


(1) $$P(t)=\sum_{i=0}^{n}P_{i}B_{i,n}(t),t\in [0,1]$$

(2) $$B_{i,n}(t)=C_{n}^{i}t^{i}{\left(1-t\right)}^{n-i}[i=0,1,\ldots ,n]$$

According to equations (1) and (2), the third-order Bezier curve calculation equation (3) can be obtained:


(3) $$B(t)={\left(1-t\right)}^{3}P_{0}+3t{\left(1-t\right)}^{2}P_{1}+3t^{2}(1-t)P_{2}+t^{3}P_{3},t\in [0,1]$$

(4) $$k(t)=\frac{\left|\dot{x_{i}}\overset{.}{y_{i}}-\overset{.}{x_{i}}\dot{y_{i}}\right|}{{\left({\dot{x_{i}}}^{2}+{\dot{y_{i}}}^{2}\right)}^{\frac{3}{2}}},[i=0,1,\ldots ,n]$$

In equation (4), k(t) is the curvature of the path point, (x(t),y(t)) is the third-order Bezier curve obstacle avoidance path, and $\dot{x}ˎ\dot{yˎ}\overset{.}{x}ˎ\overset{.}{y}$ is the first- and second-order derivative of the path point (x(t),y(t)) on the X and Y axes.

To ensure that the third-order Bezier curve curvature k(t) is smooth in the definition domain and there is no singularity, curvature smoothing constrained Bezier curve planning was used. In this planning, the first control point $P_{1}$ moves in the direction of $\vec{SP_{0}}$ and the length of $P_{0}P_{1}$ does not exceed $0.8h_{2}$ , and the second control point $P_{2}$ moves in the $\vec{P_{4}P_{3}}$ direction and the length of $P_{2}P_{3}$ does not exceed $0.8\left(w_{1}+w_{2}\right)$ . A particle swarm algorithm (eq. 5) based on the shortest path was established to solve the optimal path with the constraint that the vertical distance of point M from the horizontal plane is greater than $h_{3}$ . An adaptive adjustment factor (eq. 6) based on the inverse tangent function was established so that the particle search range decreased with the number of iterations.


(5) $$\{\begin{array}{l} v_{i}^{n+1}=\omega \times v_{i}^{n}+c_{1}\times R_{1}\times (Pbest_{i}-x_{i}^{n})+c_{2}\times R_{2}\times \left(Gbest_{i}-x_{i}^{n}\right)\\ x_{i}^{n+1}=x_{i}^{n}+r\times v_{i}^{n+1} \end{array}$$

(6) $$r=1-\tanh\frac{n}{1+n_{\max}}$$

where *i* is the particle number, *n* is the number of iterations, ω is the inertia factor, $c_{1},c_{2}$ is the learning factor of the particle, *r* is the adjustment factor, $R_{1},R_{2}$ is a random number between 0 and 1, v is the velocity of the particle, x is the position of the particle, Pbest is the historical best position of particle *i*, and Gbest is the historical best position of the particle population.

## 3 Evaluation of detection and trajectory planning

### 3.1 Evaluation of Peucedani Radix detection

For the evaluation of Peucedani Radix detection, this study used three evaluation metrics to assess the performance of the YOLOV5 network: precision, recall, and mean Average Precision (map). The Intersection over Union (IOU) is the ratio of the overlap area between the predicted bounding box and the true bounding box to the area contained in the predicted and true bounding boxes (eq. 7).


(7) $$IOU=\left(\frac{A_{1}\cap A_{2}}{A_{1}\cup A_{2}}\right)$$

where $A_{1}$ is the area of the predicted bounding box and $A_{2}$ is the area of the real bounding box.


(8) $$precision=\frac{TP}{TP+FP}$$

(9) $$recall=\frac{TP}{TP+FN}$$

where TP , FP , and FN are the number of true positive cases, false positive cases, and false negative cases, respectively.

**Figure 7:**
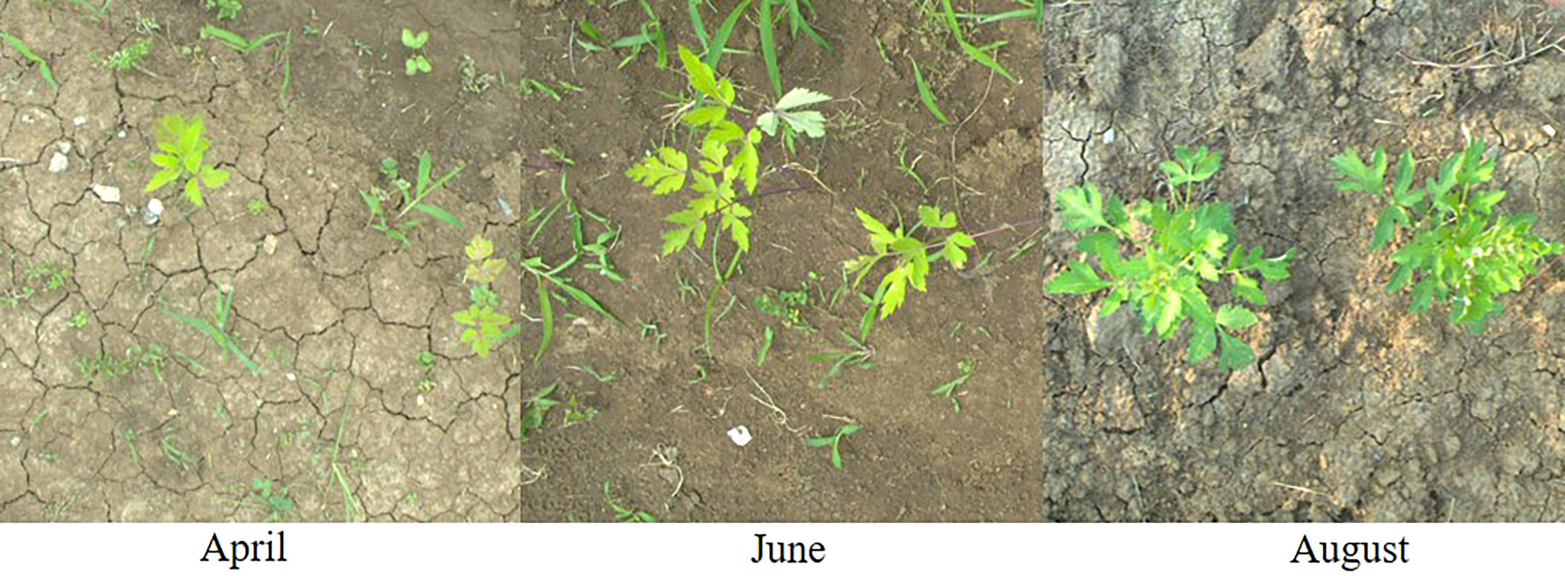
Peucedani Radix plants in different months.

The datasets of Peucedani Radix plants collected in April, June, and August were fed into the model training and validation and the validation results are shown in **Table 2**. The validation results for the June and August datasets were better than those for the April dataset. Although the precision of the April dataset was high, the recall rate was only 76.3%. There are several reasons for the poor detection results of the April dataset: compared to the June and August datasets, the number of April datasets was relatively small and the model did not produce reliable results for the extraction of Peucedani Radix features in April. In addition, as shown in **Figure 7**, Peucedani Radix plants in April were smaller and less distinctive than those in June and August. As shown in **Table 2**, the validation effect improved as Peucedani Radix grew, with an accuracy of 99.2%, recall of 91.6%, and map (IOU=0.5) of 95.8% for the August dataset when the plants were largest.

**Table 2:** Results of validation detection in April, June, and August.

Period	TP	FP	FN	Precision (%)	Recall (%)	Map (IOU=0.5)	Map (IOU=0.5:0.95)
April	720	11	224	98.5	76.3	87.8	83.1
June	1,349	20	111	98.5	92.4	95.5	91.5
August	1,327	11	122	99.2	91.6	95.8	95
All	3,399	45	454	98.7	88.2	93.8	91.1

### 3.2 Evaluation of weed split

**Figure 8:**
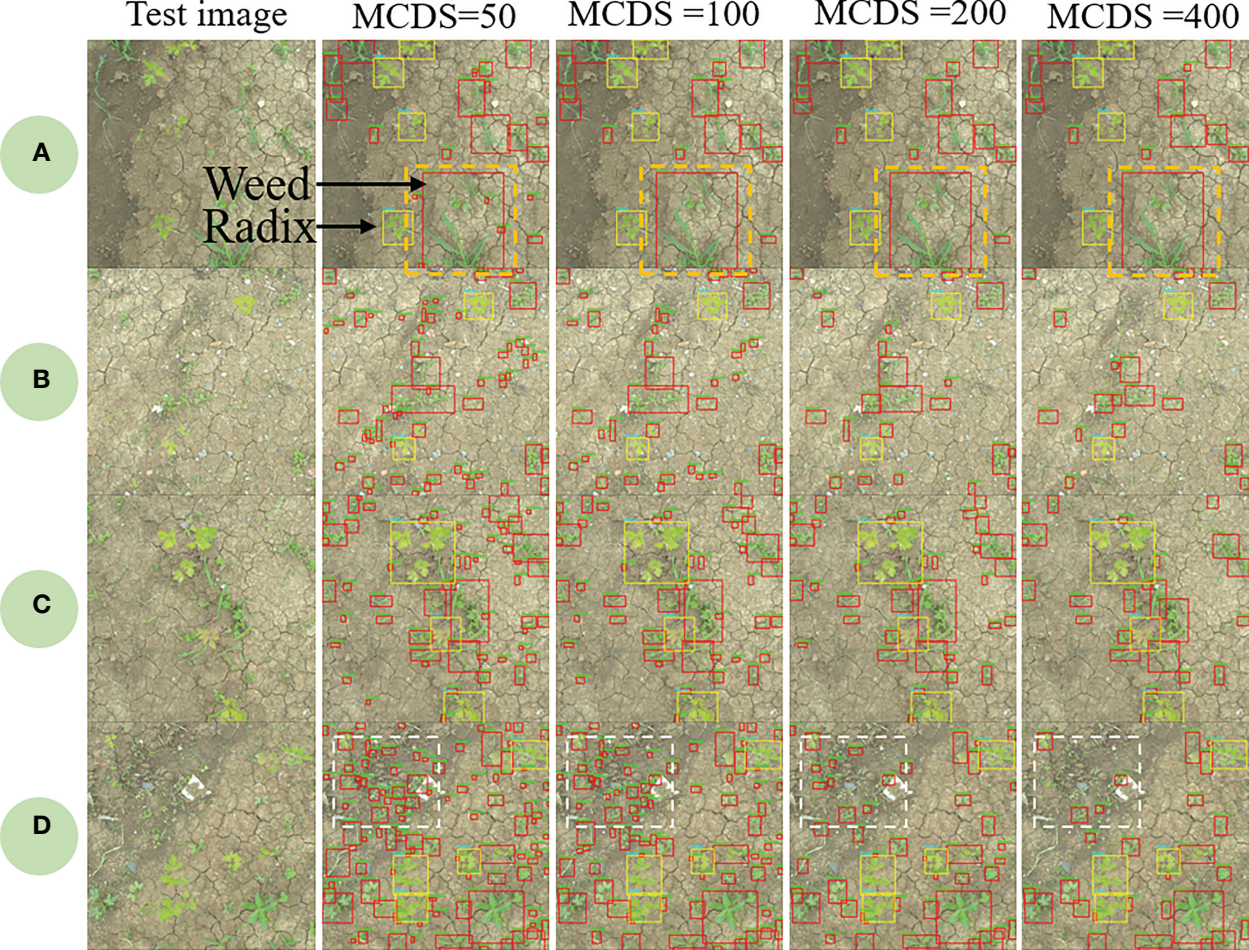
Results of weed segmentation at different minimum connected domain sizes (MCDS).

As previously described in **Figure 4**, after extracting Peucedani Radix using YOLOV5, weeds of varying sizes were segmented by the ExG feature algorithm. To verify the validity of the method, a test set of Peucedani Radix field pictures with different weed sizes and densities was used for testing. **Figure 8** shows the results of multiple images with different weed sizes and densities on the ExG feature algorithm for weed segmentation labeling. In **Figures 8A–C**, and d are the test images in order of increasing weed density. The minimum weed volume segmented by the ExG feature marker increased as the minimum connected domain size (MCDS) increased, as shown in the white boxed area in **Figure 8D**. When MCDS=50, the weed segmentation rate can attain over 95%. This means that the model can be adjusted to segment the minimum connected domain size according to the actual growth size state of the weed to achieve accurate weed identification.

To verify the development of the weed segmentation model, the weed identification model of YOLOV5 combined with ExG feature segmentation proposed in this study was compared with the YOLOV5 direct weed identification algorithm and validated. First, the dataset that was labeled with Peucedani Radix crops was secondarily labeled with all the weed samples in the dataset. The annotated dataset was enhanced in the same way. The composition of the enhanced weed Peucedani Radix dataset is shown in **Table 3**. Then, the dataset was fed into the YOLOV5 model for training. Finally, the two algorithms were tested independently using Peucedani Radix field images with different weed sizes and densities.

**Table 3:** Composition of weed dataset.

Name	Quantity (No.)	Graminaceae	Broadleaf	Sauraceae
Preferred dataset	2,347	3,472	2,889	1,236
Image augmentation	9,388	13,888	11,556	4,944
Training dataset	7,577	11,110	9,244	3,955
Test dataset	1,811	2,778	2,312	989

**Figure 9:**
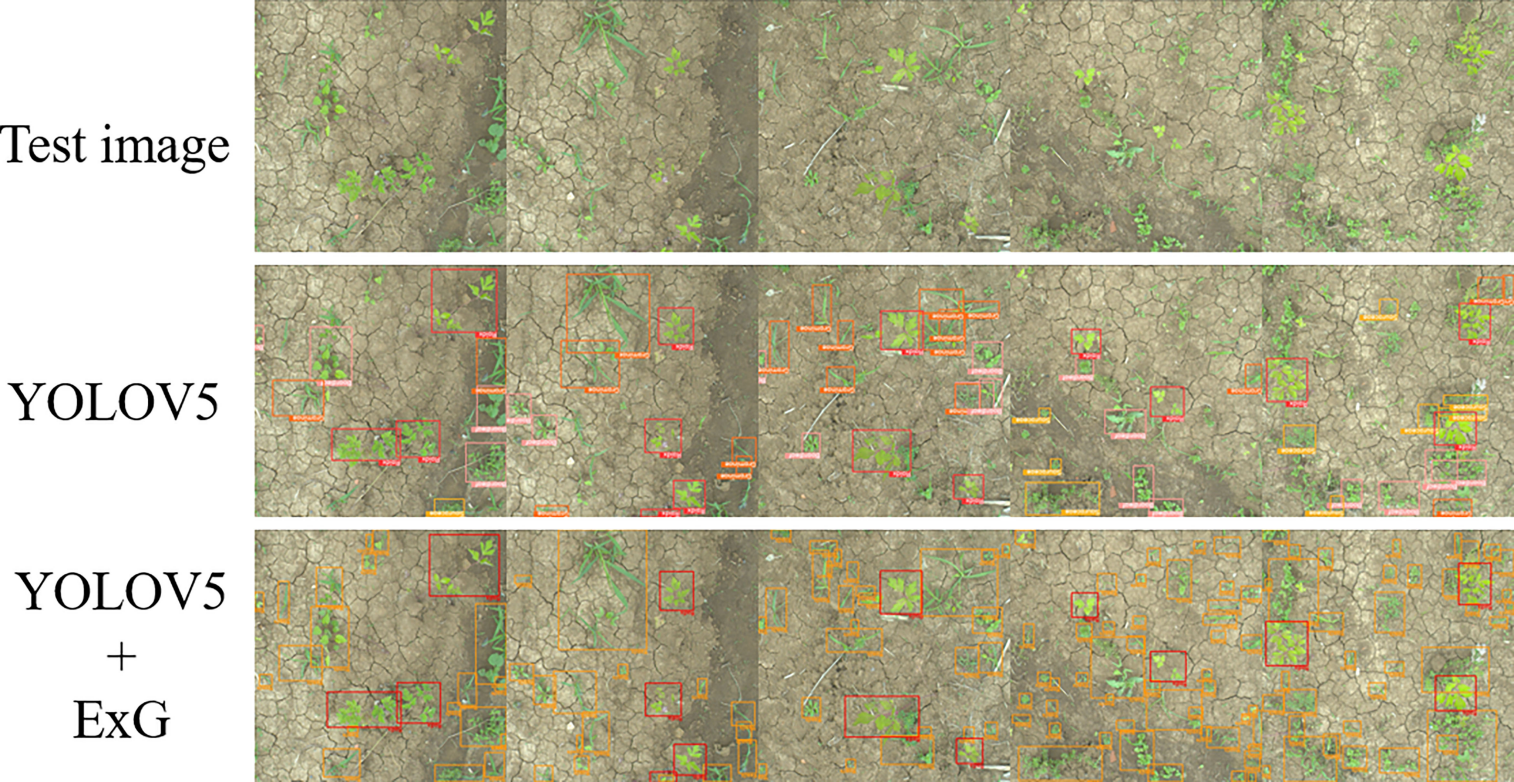
Schematic diagram comparing the effectiveness of the YOLOv5 and YOLOv5+ExG methods in identifying Peucedani Radix plants with weeds.

As shown in **Figure 9**, the five images present gradually increasing weed density from left to right. By comparing the experimental results, we found that the direct method of using YOLOV5 to identify weeds and Peucedani Radix crops could only identify some weeds with larger size and distinct features, but not all of them. However, combining YOLOV5 with ExG to first identify the Peucedani Radix plants and then perform weed segmentation allowed us to accurately segment most weeds despite the gradual increase in weed density. The strategy of combining YOLOV5 with ExG to identify Peucedani Radix plants and weeds showed superior performance compared to the direct use of YOLOV5 alone.

### 3.3 Evaluation of weed trajectory planning

**Figure 10:**
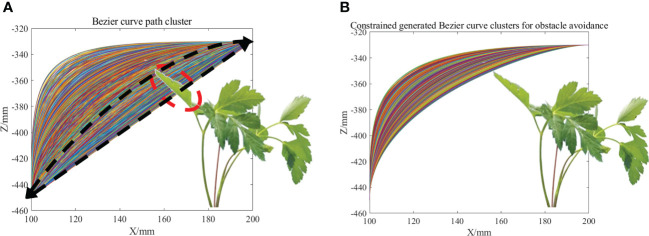
**(A)** Cluster of Bessel trajectories generated by PSO. **(B)** Cluster of seedling avoidance Bezier trajectories generated by PSO.

The simulation was performed using the proposed PSO-Bezier trajectory generation method as described previously. In the simulation, the particle swarm number was set to 5 and the maximum number of iterations to 200. The height of the sample Peucedani Radix plants was $h_{3}=150$ and trajectory planning height was $h_{1}+h_{2}=170$ . The distance between the horizontal center of Peucedani Radix and the starting point of the trajectory was set as SO=100 , where $w_{1}=60$ . The Bezier curve generated by the particle swarm is shown in **Figure 10A**. Where the Bezier trajectory (black dashed envelope) intersects with Peucedani Radix is shown using a red dashed envelope. The collision-free Bezier trajectory curve (**Figure 10B**) generated by the particle swarm was obtained by establishing the obstacle avoidance constraint through the height relationship between points M and $A_{2}$ in **Figure 6**.

**Figure 11:**
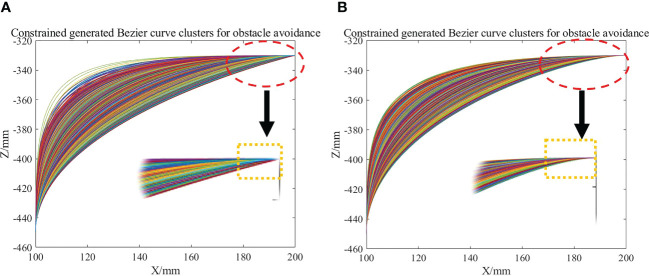
**(A)** Bezier trajectory before curvature constraint. **(B)** Bezier trajectory after curvature constraint.

As shown in the yellow dashed box in **Figure 11A**, the Bezier trajectory generated only by the height relationship between points M and $A_{2}$ shows a large change of direction at the end of the trajectory and the connection point $P_{3}$ of the horizontal movement stage, which leads to a large vibration of the robot arm when it passes through this point at high speed. After introducing the curvature constraint (eq. 4), the first control point $P_{1}$ moves in the $\vec{SP_{0}}$ direction, the length of $P_{0}P_{1}$ does not exceed $0.8h_{2}$ , the second control point $P_{2}$ moves in the direction $\vec{P_{4}P_{3}}$ , and the length of $P_{2}P_{3}$ does not exceed $0.8\left(w_{1}+w_{2}\right)$ . The effect of the curvature constraint is shown in the yellow dashed box indicated by the arrow in **Figure 11B**. Compared with **Figure 11A**, the end of the trajectory is smoother at the connection point $P_{3}$ between the end of the trajectory and the horizontal moving stage after the curvature constraint, and the vibration of the frame will be significantly reduced when the robot arm moves at high speed.

**Figure 12:**
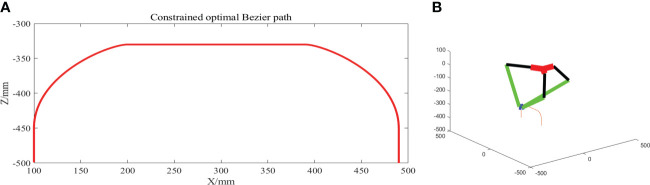
**(A)** Optimal Bezier curve path profile planned. **(B)** Schematic diagram of the inverse spatial state kinematic solution of the global obstacle avoidance trajectory.

The global obstacle avoidance trajectory planned with obstacle avoidance and curvature constraints is shown in **Figure 12A**. As shown in **Figure 12B**., the global obstacle avoidance trajectory is inverse kinematically solved by establishing the inverse kinematic inverse solution model of the parallel robotic arm in Python. The global obstacle avoidance trajectory is discretized into 100 trajectory points, and the angle that the three motors of the parallel robot arm need to rotate is obtained by solving the difference of the pose angle of the robot active arm corresponding to two adjacent trajectory points, and the robot arm end-effector moves along the planned trajectory through equal interpolation.

## 4 Weeding experiments

### 4.1 Introduction of the experimental system

**Figure 13:**
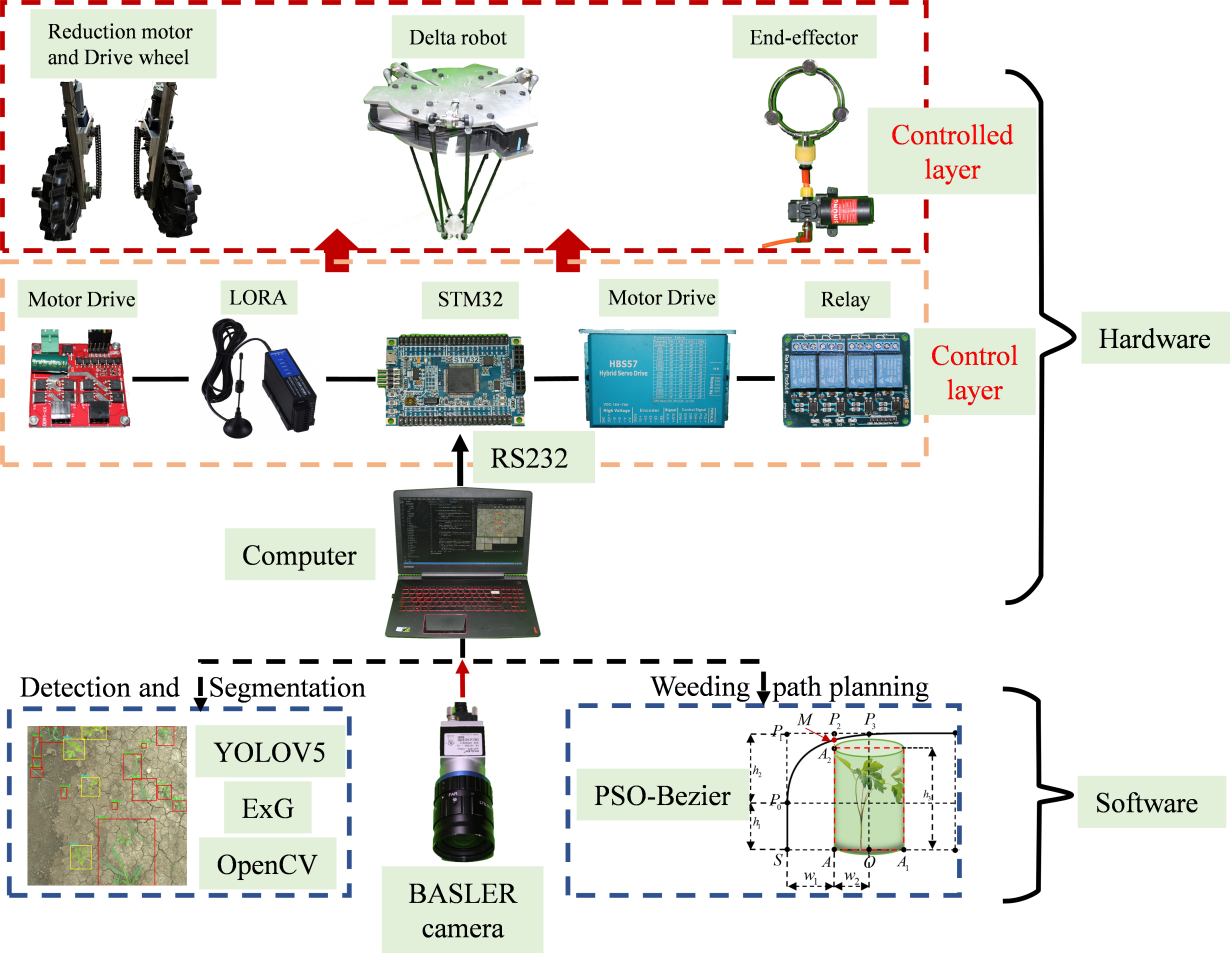
System of the Peucedani Radix weeding robot.

The machine used for the Peucedani Radix weeding experiment consisted mainly of an industrial camera, a computer, a control layer device, and a controlled layer device, as shown in **Figure 13**. The control layer contained the microcontroller STM32F407, the LORA communication module, the motor driver, and the relays. The controlled layer contained geared motors and drive wheels, Delta parallel manipulators, and end spray actuators. The Delta parallel manipulator was designed and made by our team (Zhang et al., 2023) and the repeat positioning accuracy was 4 mm.

In **Figure 13**, the robot first captures images of the crop and weeds on the monopoly using a camera mounted at an angle of 90° to the horizontal plane, and the computer identifies and locates the positions of the Peucedani Radix plants and weeds in real time. Subsequently, the computer performs PSO-Bezier trajectory planning for the end-effector of the robot arm based on the position and morphological parameters of the Peucedani Radix plants and the positions of the weeds. Then, the computer sends commands to the STM32 microcontroller to control the robot to advance a fixed distance based on the horizontal fixed distance between the origin of the camera coordinate system and the origin of the robot arm coordinates. Finally, the computer discretizes the planned trajectory and sends it to the STM32 microcontroller in sequence through the serial port. After receiving the signal from the serial port, the STM32 microcontroller generates an interrupt and the number of pulses required for motor rotation in the interrupt service program. When the robot arm runs through all trajectory points and reaches above the weeds, the relay is activated, and the end-effector pump starts working to spray the herbicide. Through these steps, the robot achieves the function of spraying herbicide with precise seedling avoidance.

All algorithms were executed on a portable computing device (Lenovo) equipped with an Intel i5-7300HQ processor and 16 GB of RAM, operating on a Windows 10 64-bit system. The Peucedani Radix detection algorithm was implemented based on a modification of the YOLOV5 code open-source library (https://github.com/ultralytics/yolov5). The weed ExG feature segmentation algorithm was implemented based on a modification of the open-source Computer Vision library (OpenCV, https://opencv.org/). The PSO-Bezier optimal trajectory generation algorithm for seedling avoidance and weeding was designed and written by our team and deployed using Python. The STM32F407 hardware was programmed using the official firmware library (https://stmicroelectronics.com.cn) and the relevant code was written in the MDK compiler using the C language.

### 4.2 Results of weeding experiments

**Figure 14:**
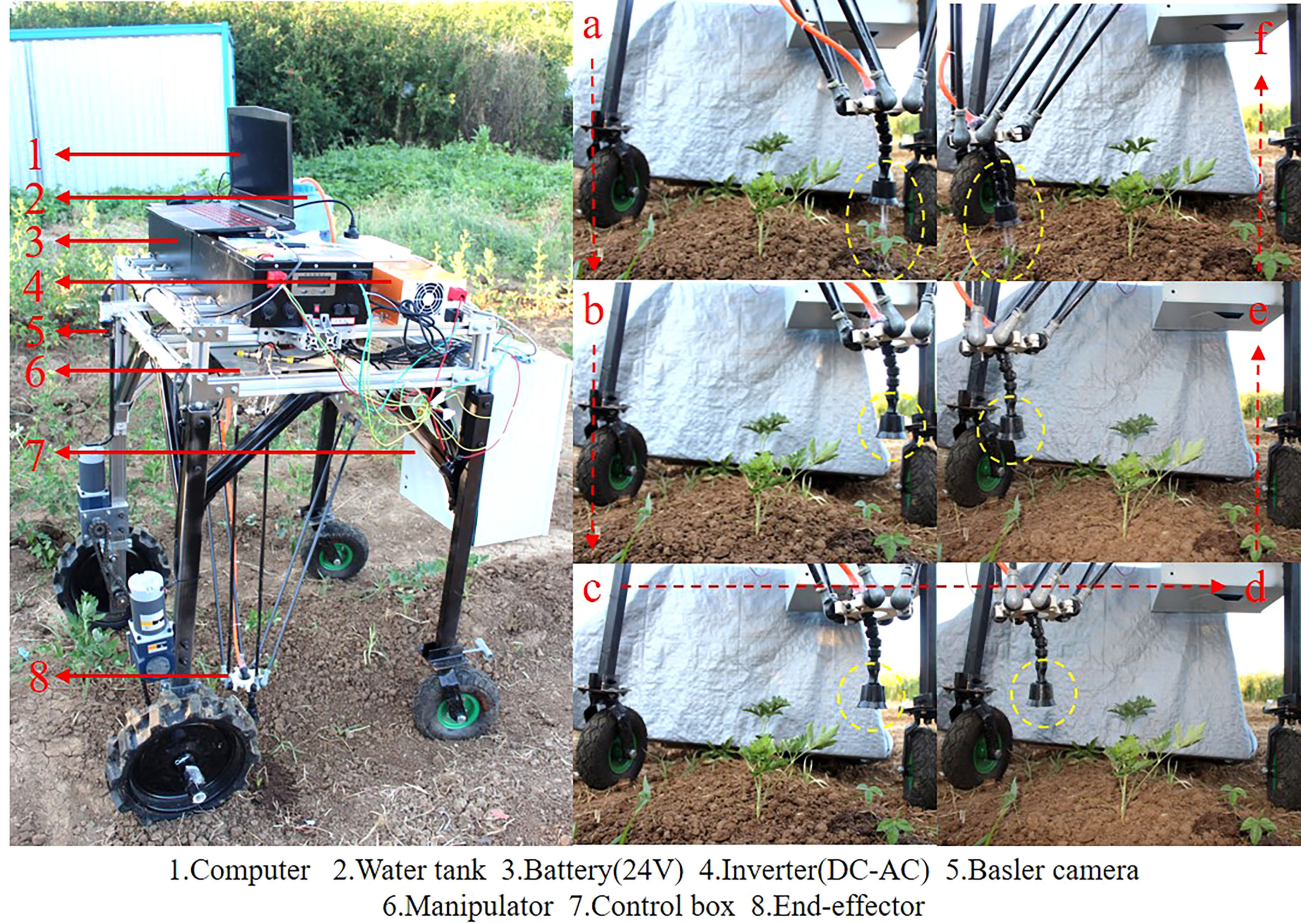
Agricultural robot field weeding experiment site.

The weeding experiment was conducted on a sunny day in August 2022 at Nongcui Garden, Anhui Agricultural University, Anhui Province, China (**Figure 14**). In the experiment, Peucedani Radix was first identified by the YOLOV5 algorithm and the weeds were then segmented by the ExG feature segmentation algorithm to obtain the Peucedani Radix size parameters and the corresponding coordinates of Peucedani Radix and weeds. The trajectory of the manipulator arm end avoidance was generated by PSO-Bezier and the joint rotation angle of the parallel manipulator arm at each step of the interpolation was obtained by inverse solution of the kinematics of the avoidance trajectory by aliquot interpolation. The angle of joint rotation was converted into motor operation drive parameters and sent to the STM32 microcontroller *via* RS232 communication, which controlled the motor driver and drove the equipment to the specified position. The end-effector started spraying herbicide when it reached above the weed to complete a spraying operation. Fifty plants were selected for 100 spraying operations to verify the accuracy of the algorithm and the results were as follows:

(1) A total of 161 weeds were successfully and accurately sprayed out of 100 spraying operations, with 39 weeds not successfully sprayed; therefore, the success rate of accurate herbicide spraying was 80.5%.
(2) In the precision spraying operation, the end-effector made four collision contacts with the Peucedani Radix plants when moving according to the generated PSO-Bezier trajectory. Therefore, the success rate of seedling avoidance for the PSO-Bezier trajectory motion of the end-effector was 96%.
(3) The failure of agricultural robots to accurately spray herbicides on weeds was partly due to failure in accurately identifying Peucedani Radix and weeds, and partly due to failure in accurately locating the weeds.
(4) The main reason for the collision contact between the end-effector of the agricultural robot and the Peucedani Radix plants was the differing heights of Peucedani Radix plants and the variable height of the terrain.

The results showed that the time to identify and locate Peucedani Radix and weeds in one frame was 0.75 s on average. The average time to generate the end-effector PSO-Bezier seedling avoidance motion trajectory was 0.35 s. The end-effector movement time increased with the linear distance between the weeds at the ends of the trajectory. The average time for the end-effector to execute the planned PSO-Bezier trajectory was 2.8 s when the weed linear spacing was 30 cm. In summary, the total time for precision herbicide spraying by the developed agricultural robot was 3–5 s, meaning that it takes an average of 2 s per weed to accurately spray herbicide. Therefore, the proposed method for precise seedling avoidance spraying of herbicides can be effectively applied to an agricultural robot platform.

## 5 Discussion

In the validation experiments of Peucedani Radix recognition, the accuracy of early Peucedani Radix recognition was relatively low because the dataset of April Peucedani Radix plants was small and plant morphology was not obvious compared with June and August. To improve this problem, we plan to add more images to the April Peucedani Radix plant dataset. In the ExG feature segmentation weed experiment, we achieved over 95% accuracy of weed segmentation at MCDM=50. In addition, through comparison experiments, the YOLOV5 method proposed in this study for identifying Peucedani Radix combined with ExG feature segmentation of weeds was able to maintain a higher recognition accuracy under different weed density conditions compared to the YOLOV5 direct weed recognition method. In particular, the ability to segment weeds with smaller targets indicates that the method is robust for weed recognition. Compared with the maize and weed detection algorithm proposed by Quan et al. (2022), the method proposed in this study eliminates the weed labeling work and significantly reduces the workload of crop and weed detection. However, as shown in the orange dashed box in **Figure 8A**, the segmented connected domain becomes larger when weeds are present in adhesion, which causes the center of the connected domain morphology to deviate from the center of the single weed morphology, thus leading to ineffective spraying of herbicides onto the weed foliage during subsequent spraying. To solve this problem, in the future, we will explore how to extract single weeds based on the weed skeleton line based on the ExG segmentation to achieve the accurate positioning of single weed centroids.

In the PSO-Bezier trajectory simulation experiments, the trajectory profile generation time was only 0.35 s. Moreover, our proposed end-effector motion trajectory can save 10–15% of the motion distance with the same start and end points compared to the Lamé3 type motion trajectory proposed by Yang et al. (2021), which effectively reduces energy consumption. This demonstrates the superior performance of our algorithm. In addition, our proposed PSO-Bezier trajectory can be adaptively parameterized according to plant characteristics, and this adjustment can enable this trajectory to be used for weeding operations of other important cash crops such as tomatoes and eggplants. However, in our study we idealized a fixed height of the Peucedani Radix plant, which in practice will lead to collisions between the end-effector and some of the taller Peucedani Radix seedlings, thus causing some damage to the Peucedani Radix crop. The reason for this phenomenon is that the camera cannot effectively obtain the exact height of the Peucedani Radix plants, a disadvantage of the camera capturing the ground image vertically. To improve this problem, we plan to use an RGB-D (Xu et al., 2017) depth camera to combine depth and image information to obtain information such as height and pose of Peucedani Radix plants.

**Figure 15:**
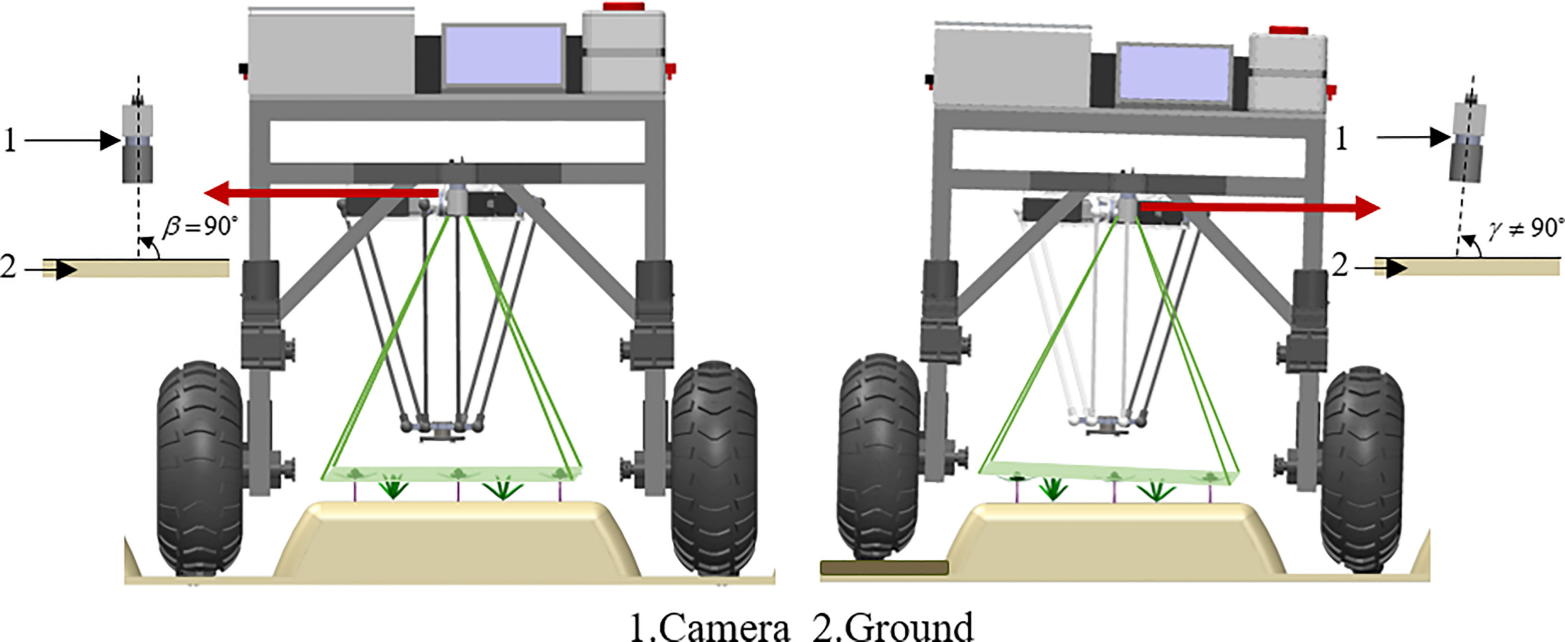
Reasons for the positioning error in camera recognition.

In the actual trial, a total of 39 weed plants were not successfully sprayed with herbicide. This was attributed to two main reasons:

(1) The actual experiment was conducted using Peucedani Radix plants in August, and the weather on the day of the experiment was sunny with sufficient sunlight at noon. The overexposure of direct sunlight on the foliage of some of the Peucedani Radix plants and weeds caused serious loss of color features of Peucedani Radix in the images, and the Peucedani Radix plants and weeds could not be identified accurately. There were eight times that the Peucedani Radix plants and weeds could not be identified in the experiment. To improve this problem, in the future, we will take field images at midday and add overexposed images to increase the diversity of our samples to increase the generalization performance of the model. In addition, we will also explore the use of shades or creation of a stable lighting environment to improve the recognition accuracy of Peucedani Radix plants and weeds.
(2) Due to the linear distance between the camera coordinate system and the origin of the parallel robot arm coordinate system, the agricultural robot platform needs to travel a fixed distance for accurate spraying operation after the camera identifies the Peucedani Radix and weeds. In this process, ground topography has a large influence on the Peucedani Radix and weed positioning. As shown in **Figure 15**, the ground is not flat and the camera plane is not parallel to the ground, which leads to a large deviation in positioning and makes it difficult for the nozzle at the end of the robot arm to accurately locate the weeds. Therefore, in the future, we will explore the use of a depth camera to identify and locate crops and weeds, and measure the deviation of the image position from the actual position by the angle A between the camera plane and the monopoly plane through the on-board altitude sensor, which can be used to compensate the position to achieve accurate spray positioning.

In summary, in this study, we applied the YOLOV5 deep learning network with ExG feature segmentation to a Peucedani Radix crop and weed detection system for the first time. We transformed crop and weed recognition into a binary classification problem, thus avoiding tedious weed labeling and improving the overall recognition accuracy, especially for small target weeds. In addition, our proposed PSO-Bezier weed avoidance trajectory better incorporated the biological characteristics of the Peucedani Radix plants compared to existing trajectories, saving the robot arm 10–15% of the movement distance, which will significantly reduce the energy consumption of the robot’s work in practical applications. However, there remain limitations to our research, such as the inability to accurately identify Peucedani Radix plants and weeds under high exposure conditions and the lack of accuracy in herbicide spraying due to changes in the robot’s body position. In future work, we plan to address these issues by expanding the image dataset for high exposure conditions, using RGB-D depth cameras, and building a position error compensation model.

## 6 Conclusions

An intelligent agricultural robot was designed to accurately spray weeds in Peucedani Radix fields with herbicide. The agricultural robot identified Peucedani Radix plants and weeds in the field using YOLOV5 combined with the ExG segmentation algorithm, used the PSO-Bezier algorithm to plan the optimal seedling avoidance spraying path, and executed this based on Peucedani Radix growth parameters obtained by identification. We evaluated the performance of the agricultural robots and algorithms through simulation validation combined with real-world operations. The following specific conclusions can be drawn:

(1) The overall precision and recall of Peucedani Radix detection in the Peucedani Radix field environment used for the experiment were 98.7% and 88.2%, respectively, and the map was 93.8% at IOU=0.5. When the MCDS was 50, the ExG feature segmentation algorithm can achieve 95% segmentation rate for weeds. In addition, the study evaluated the results of Peucedani Radix testing at different periods and found that the best results were obtained in August, when the plants were the largest.
(2) The study introduced a novel scheme for precise herbicide spraying by seedling avoidance, which used the PSO-Bezier algorithm to effectively achieve precise herbicide spraying in combination with the growth characteristics of Peucedani Radix. Compared with the Lamé3 transition trajectory, the trajectory generated by the arm running the PSO-Bezier algorithm can reduce movement distance by 10–15%, which effectively reduces the energy consumption required for arm operation.
(3) The effectiveness of the proposed algorithm was verified in actual field weeding experiments. The results showed that the success rate of field precision seedling avoidance herbicide spraying was 80.5%, the collision rate between the robotic arm end-effector and Peucedani Radix was 4%, the average detection time of the proposed algorithm for weeds and Peucedani Radix was 0.75 s per image, the average generation time of a single PSO-Bezier motion trajectory was 0.35 s, and the robotic arm could precisely spray a single weed an average of every 2 s.

Overall, the herbicide spraying method proposed in this study for precise seedling avoidance in a Peucedani Radix field was effective. The detection time of the proposed algorithm for weeds and the generation time of weeding trajectories are reasonable for agricultural robots. Also, this research can be widely applied to weed control in fields of tomato, eggplant, and other important crops worldwide. However, uneven terrain and large areas of weeds sticking between monopolies negatively impact weed center point positioning and accurate spraying. In addition, the precise timing of herbicide spraying in the field was not satisfactory. In future work, we will continue to study the above-mentioned points, improve the productivity of agricultural robots based on actual weeding operation scenarios, and contribute to the development of agricultural automation and precision agriculture.

## Data availability statement

The raw data supporting the conclusions of this article will be made available by the authors, without undue reservation.

## Author contributions

ZX designed the sorting robot structure, control system and experiments, conducted the data analyses, and drafted the manuscript. CC provided financial support for the article, designed the framework of the study, and acts as the corresponding author. WZ helped to design the framework of the study. LK implemented the software and helped to process the figures and tables. QK and AM helped in the design of the mechanical structure and prototype commissioning. DW helped in the design of the control system and tests. XW helped prepare materials and tests. All authors contributed to the article and approved the submitted version.

## References

[B1] Ahmad A. Saraswat D. Aggarwal V. Etienne A. Hancock B. (2021). Performance of deep learning models for classifying and detecting common weeds in corn and soybean production systems. Comput. Electron. Agric. 184, 106081. doi: 10.1016/j.compag.2021.106081

[B2] Chang H.-M. But P.-P. H. Yao S.-C. Wang L.-L. Yeung S. C.-S. (1986). Pharmacology and Applications of Chinese Materia Medica: (Volume I). WORLD SCIENTIFIC doi: 10.1142/0284

[B3] Chavan T. R. Nandedkar A. V. (2018). AgroAVNET for crops and weeds classification: a step forward in automatic farming. Comput. Electron. Agric. 154, 361–372. doi: 10.1016/j.compag.2018.09.021

[B4] Dos Santos Ferreira A. Freitas D. M. da Silva G. G. Pistori H. Folhes M. T. (2017). Weed detection in soybean crops using ConvNets. Comput. Electron. Agric. 143, 314–324. doi: 10.1016/j.compag.2017.10.027

[B5] Hamuda E. Mc Ginley B. Glavin M. Jones E. (2017). Automatic crop detection under field conditions using the HSV colour space and morphological operations. Comput. Electron. Agric. 133, 97–107. doi: 10.1016/j.compag.2016.11.021

[B6] Hasan A. M. Sohel F. Diepeveen D. Laga H. Jones M. G. (2021). A survey of deep learning techniques for weed detection from images. Comput. Electron. Agric. 184, 106067. doi: 10.1016/j.compag.2021.106067

[B7] Li N. Chen Z. Zhang X. Liu X. (2021). An ultra-fast bi-phase advanced network for segmenting crop plants from dense weeds. Biosyst. Eng. 212, 160–174. doi: 10.1016/j.biosystemseng.2021.10.008

[B8] Li Y. Guo Z. Shuang F. Zhang M. Li X. (2022). Key technologies of machine vision for weeding robots: a review and benchmark. Comput. Electron. Agric. 196, 106880. doi: 10.1016/j.compag.2022.106880

[B9] Özlüoymak Ö.B. (2022). Development and assessment of a novel camera-integrated spraying needle nozzle design for targeted micro-dose spraying in precision weed control. Comput. Electron. Agric. 199, 107134. doi: 10.1016/j.compag.2022.107134

[B10] Partel V. Kakarla S. C. Ampatzidis Y. (2019). Development and evaluation of a low-cost and smart technology for precision weed management utilizing artificial intelligence. Comput. Electron. Agric. 157, 339–350. doi: 10.1016/j.compag.2018.12.048

[B11] Pharmacopoeia C. (2010). The state pharmacopoeia commission of PR China Vol. 3 (Beijing: China Medical Science).

[B12] Picon A. San-Emeterio M. G. Bereciartua-Perez A. Klukas C. Eggers T. Navarra-Mestre R. (2022). Deep learning-based segmentation of multiple species of weeds and corn crop using synthetic and real image datasets. Comput. Electron. Agric. 194, 106719. doi: 10.1016/j.compag.2022.106719

[B13] Quan L. Feng H. Lv Y. Wang Q. Zhang C. Liu J.. (2019). Maize seedling detection under different growth stages and complex field environments based on an improved faster r–CNN. Biosyst. Eng. 184, 1–23. doi: 10.1016/j.biosystemseng.2019.05.002

[B14] Quan L. Jiang W. Li H. Li H. Wang Q. Chen L. (2022). Intelligent intra-row robotic weeding system combining deep learning technology with a targeted weeding mode. Biosyst. Eng. 216, 13–31. doi: 10.1016/j.biosystemseng.2022.01.019

[B15] Redmon J. Farhadi A. (2018). YOLOv3: An Incremental Improvement. doi: 10.48550/arXiv.1804.02767

[B16] Strothmann W. Ruckelshausen A. Hertzberg J. Scholz C. Langsenkamp F. (2017). Plant classification with in-field-labeling for crop/weed discrimination using spectral features and 3d surface features from a multi-wavelength laser line profile system. Comput. Electron. Agric. 134, 79–93. doi: 10.1016/j.compag.2017.01.003

[B17] Sujaritha M. Annadurai S. Satheeshkumar J. Sharan S. K. Mahesh L. (2017). Weed detecting robot in sugarcane fields using fuzzy real time classifier. Comput. Electron. Agric. 134, 160–171. doi: 10.1016/j.compag.2017.01.008

[B18] Tang J. Wang D. Zhang Z. He L. Xin J. Xu Y. (2017). Weed identification based on K-means feature learning combined with convolutional neural network. Comput. Electron. Agric. 135, 63–70. doi: 10.1016/j.compag.2017.01.001

[B19] The State Pharmacopoeia, C. (2010). The state pharmacopoeia commission of PR China Vol. 3 (Beijing: China Medical Science).

[B20] The State Pharmacopoeia Commission of P.R. China (1997). Pharmacopoeia of the people’s republic of China (Beijing, China: Chemical Industry Press).

[B21] Utstumo T. Urdal F. Brevik A. Dørum J. Netland J. Overskeid Ø.. (2018). Robotic in-row weed control in vegetables. Comput. Electron. Agric. 154, 36–45. doi: 10.1016/j.compag.2018.08.043

[B22] Villette S. Maillot T. Guillemin J. P. Douzals J. P. (2021). Simulation-aided study of herbicide patch spraying: influence of spraying features and weed spatial distributions. Comput. Electron. Agric. 182, 105981. doi: 10.1016/j.compag.2020.105981

[B23] Villette S. Maillot T. Guillemin J. P. Douzals J. P. (2022). Assessment of nozzle control strategies in weed spot spraying to reduce herbicide use and avoid under-or over-application. Biosyst. Eng. 219, 68–84. doi: 10.1016/j.biosystemseng.2022.04.012

[B24] Xu X. Li Y. Wu G. Luo J. (2017). Multi-modal deep feature learning for RGB-d object detection. Pattern Recogn. 72, 300–313. doi: 10.1016/j.patcog.2017.07.026

[B25] Yang H. Chen L. Ma Z. Chen M. Zhong Y. Deng F.. (2021). Computer vision-based high-quality tea automatic plucking robot using delta parallel manipulator. Comput. Electron. Agric. 181, 105946. doi: 10.1016/j.compag.2020.105946

[B26] Zhang X. Wu Z. Cao C. Luo K. Qin K. Huang Y.. (2023). Design and operation of a deep-learning-based fresh tea-leaf sorting robot. Comput. Electron. Agric. 206, 107664. doi: 10.1016/j.compag.2023.107664

[B27] Zheng Y. Zhu Q. Huang M. Guo Y. Qin J. (2017). Maize and weed classification using color indices with support vector data description in outdoor fields. Comput. Electron. Agric. 141, 215–222. doi: 10.1016/j.compag.2017.07.028

[B28] Zou K. Chen X. Wang Y. Zhang C. Zhang F. (2021). A modified U-net with a specific data argumentation method for semantic segmentation of weed images in the field. Comput. Electron. Agric. 187, 106242. doi: 10.1016/j.compag.2021.106242

